# 

**Published:** 1895-09

**Authors:** 


### CONTENTS FOR SEPTEMBER, 1895.

ORIGINAL COMMUNICATIONS. PAGE.

Observations on Rectal Surgery. By J. M. MATHEWS, M. D ........ 129
A Study of the Origin and Nature of Pain. By HERMAN G. MATZINGER, M. D ........ 137
Care and Treatment of Old People. By SAMUEL G. DORR, M. D ........ 144
Abuse and Use of Atropia in Eye Treatment. By RICHARD H. SATTERLEE, M. D ........ 149

PROGRESS IN MEDICAL SCIENCE.

Ophthalmology ........ 150
Physiological Chemistry ........ 156
Pediatrics ........ 159
State Medicine, Public Health, Hygiene and Bacteriology ........ 162
State Medical Examinations ........ 165

SOCIETY PROCEEDINGS.

Medical Society of the County of Chautauqua ........ 172

EDITORIAL.

The Erie County Hospital ........ 174
Topics of the Month ........ 176

Obituary ........ 177
Personal ........ 178, 204
Notes from Chautauqua County ........ 179
What Our Contemporaries Think of Us ........ 181
Reviews ........ 195
Literary ........ 204
Miscellany ........ 206

